# Mass spectrometry-based lipidomics to explore the biochemical effects of naphthalene toxicity or tolerance in a mouse model

**DOI:** 10.1371/journal.pone.0204829

**Published:** 2018-10-01

**Authors:** Sheng-Han Lee, Si-Han Hong, Chuan-Ho Tang, Yee Soon Ling, Ke-Han Chen, Hao-Jan Liang, Ching-Yu Lin

**Affiliations:** 1 Institute of Environmental Health, College of Public Health, National Taiwan University, Taipei, Taiwan; 2 National Museum of Marine Biology and Aquarium, Pingtung, Taiwan; 3 Institute of Marine Biodiversity and Evolutionary Biology, National Dong Hwa University, Pingtung, Taiwan; 4 Graduate Institute of Toxicology, College of Medicine, National Taiwan University, Taipei, Taiwan; Weill Cornell Medical College in Qatar, QATAR

## Abstract

Naphthalene causes mouse airway epithelial injury. However, repeated exposures of naphthalene result in mouse airway tolerance. Previous results showed that toxicity or tolerance was correlated with changes of phosphorylcholine-containing lipids. In this study, a mass spectrometry-based lipidomic approach was applied to examine the effects of naphthalene-induced injury or tolerance in the male ICR mice. The injury model was vehicle x 7 plus 300 mg/kg naphthalene while the tolerant one was 200 mg/kg daily x 7 followed by 300 mg/kg naphthalene on day 8. The lung, liver, kidney, and serum samples were collected for profiles of phosphorylcholine-containing lipids including phosphatidylcholines (PCs) and sphingomyelins (SMs). A partial least-square-discriminate analysis model showed different lung phosphorylcholine-containing lipid profiles from the injured, tolerant, and control groups. Perturbation of diacyl-PCs and plasmenylcholines may be associated with enhanced membrane flexibility and anti-oxidative mechanisms in the lungs of tolerant mice. Additionally, alterations of lyso-PCs and SMs may be responsible for pulmonary dysfunction and inflammation in the lungs of injured mice. Moreover, serum PC(16:0/18:1) has potential to reflect naphthalene-induced airway injuries. Few phosphorylcholine-containing lipid alterations were found in the mouse livers and kidneys across different treatments. This study revealed the changes in lipid profiles associated with the perturbations caused by naphthalene tolerance and toxicity; examination of lipids in serum may assist biomarker development with the potential for application in the human population.

## Introduction

Naphthalene, the most common polycyclic aromatic hydrocarbon (PAH), is present in both air and groundwater from a variety of sources, such as industrial plants, vehicle traffic, and forest fires [1, 2]. In addition, moth repellents, cigarette smoking, deodorant and furniture also release certain amounts of naphthalene [3]. Studies have shown that naphthalene can be detected in various tissues and organs from farm animals and human bodies [4–7].

Previous studies showed naphthalene induced respiratory toxicity in a mouse model. After mice received a single dose of naphthalene (200 mg/kg, i.p.), non-ciliated, bronchiolar epithelial (Clara) cells in the airway were injured, whereas there was no obvious tissue damage in the liver or kidney [8]. Another study further illustrated naphthalene-induced intracellular changes, including membrane bleb formation, a swollen smooth endoplasmic reticulum, swollen mitochondria with granular matrices, cytoskeletal filament rearrangements, and increased membrane permeability on injured terminal bronchiolar Clara cells [9].

In contrast to the severe Clara cell injury in the mouse lungs after single dose of naphthalene exposure, multiple daily repeated doses of naphthalene showed tolerability for a higher dose of naphthalene treatment. The experimental results from O’Brien et al. showed that after repeated exposure of naphthalene (200 mg/kg, i.p.) for seven days, the mouse lung loses susceptibility to acute injury, whereas these animals could eventually tolerate a higher challenge dose (300 mg/kg) compared to the injury model, which was administered with vehicle for seven days, followed by a single challenging dose of naphthalene. Similar results were also found in a mouse inhalation model [10]. The glutathione (GSH) pool and enzymes for GSH synthesis were suggested to partially explain the observed phenomenon of naphthalene tolerance [11, 12].

Our previous studies have applied ^1^H NMR-based metabolomics to study metabolic perturbation in the lungs of mice after a single dose or repeated doses of naphthalene exposure [13–15]. An imbalanced energy supply, lipid peroxidation, and loss of membrane structure components (especially in phosphorylcholine-containing lipids) in the mouse respiratory system may be related to naphthalene toxicity, while induction of detoxification mechanisms was associated with naphthalene tolerance.

A previous study also compared metabolic effects among various organs including the lung, liver, and kidney in naphthalene toxicity or tolerance mouse models [14]. Fewer metabolic responses were observed in mouse livers and kidneys than lungs despite treatments. The balance between generation of reactive metabolites and the cellular capacity for detoxication in the liver and kidney may explain the lack of obvious cell injury in those organs after naphthalene treatment. Since NMR could only provide limited ability to discriminate between different lipid species, a greater sensitivity and selectivity mass spectrometry (MS) technique for lipid analysis is essential for understanding the lipid perturbation associated with naphthalene toxicity or tolerance.

Lipidomics, a branch of metabolomics, has become a popular research interest in the post-genomic revolution and systems biology [16]. Phosphorylcholine-containing lipids, which are composed of various species of phosphatidylcholines (PCs) and sphingomyelins (SMs), are a major component of cellular membranes. PCs are the most abundant among phospholipids. Moreover, a study has documented that PCs are an effective source of choline (an essential nutrient), a phospholipid cell membrane builder, an osmotic protectant and an osmoregulator of the cell [17]. SMs are a kind of sphingolipids, which share the same head group (phosphorylcholine) with PCs in a long-chain phosphorylcholine bases, whereas the remaining amide-linked acyl chain backbone is different from the PCs. SMs not only play an important role in the membrane microenvironments (such as caveolae and lipid rafts) [18] but also in the precursor pool of second messengers for cellular signal transduction [19]. Previously, our laboratory developed a validated liquid chromatography-tandem MS platform to monitor the alterations of different phosphorylcholine-containing lipids and apply them in studying pulmonary toxicity [20, 21].

In this study, we aimed to examine changes of phosphorylcholine-containing lipids to understand the effects of repeated or single exposures of naphthalene in tolerant or injury models, respectively, in order to suggest the potential mechanisms of naphthalene toxicity. The target organ (lung), non-target organs (liver and kidney), and serum were examined with a goal of suggesting potential markers for respiratory injury in the human population.

## Materials and methods

### Animals

Biological samples were taken from the same animals in previous study [14]. Male ICR mice (6~7 weeks old) were purchased from BioLASCO (Taiwan) and acclimated for one week prior to the experiments. The mice were housed in plastic cages on chip bedding and maintained in a conditioned environment with temperature 20~ 25°C, 35~ 50% relative humidity, and a 12-h light/dark cycle. Animals had free access to water and certified rodent chow (LabDiet 5001, LabDiet Inc, USA). All animal experiments were approved by the National Taiwan University’s animal care and use committee (Permit Number: 20090069).

### Naphthalene treatment

The experimental animals were randomly divided into three groups (*n* = 2 per group for histopathology; *n* = 6 per group for lipidomics analysis): repeated dose treatment (tolerant model), single dose treatment (injury model or non-tolerant model), and the control group. The repeated dose group was intraperitoneally administered 200 mg/kg naphthalene daily for seven days and then administered a challenged dose (300 mg/kg naphthalene) on the eighth day. The single dose group was intraperitoneally administered vehicle (olive oil) daily for seven days, followed by administration of a challenged dose (300 mg/kg naphthalene) on the eighth day. The control group was intraperitoneally administered with olive oil daily for eight days. At 24 h after a challenge-dose, the animals (weight ranged from 30–35 g) were sacrificed by Avertin (2,2,2-tri-bromoethanol 250 mg/ kg of body weight). Initially, whole blood samples were collected via cardiac puncturing. Following, whole blood was centrifuged for 15 min at 1,600 rpm, 4°C. Supernatant (serum) samples were collected and stored at –80°C until further analysis. The lung, liver, and kidney were removed and rinsed with PBS and then frozen immediately with liquid N_2_ and stored at -80°C until further analysis. One mouse of the repeated dose group was dead, thus, n = 5 for that group.

### Histopathology

After treatment and animal sacrifice, the tracheas of mice were cannulated. The thoraxes were unfolded by diaphragmatic incision, and the process of tracheal infusion was carried out without removing the lungs from the chest [22]. The collapsed lungs were immersed in 4% formaldehyde through tracheal infusion and stayed for 24 h at 30 cm height fluid pressure. After the collapsed lung was completely filled with 4% formaldehyde, it was removed from the chest and immersed in 4% formaldehyde overnight. Xylene was used to purge tissues samples (including lung, liver, and kidney) before embedding in paraffin wax. After haematoxylin and eosin staining, tissues samples were cut into serial sections (5 μm thickness). The serial sections were prepared for the light microscopic examination.

### Sample preparation for lipid analysis

The frozen tissues (lung, liver, and kidney) were homogenized using a liquid N_2_-cooled mortar and pestle first, after which they were lyophilized overnight for lipid extraction. The dry tissue and serum samples were extracted based on Folch’s protocol [23] with appropriate modifications [20]. The 1.0 mg of tissue powder or 10 μl of serum samples were transferred to an Eppendorf tube with 600 μl extraction solvent (chloroform: methanol: NaCl = 8: 4: 3). After the mixture was vortexed and went through 10 minutes centrifugation (11,000 rpm at 10°C), the mixture was divided into an upper and a lower layer. The entire lower layer (lipids) was transferred and evaporated at room temperature. These lipid extracts were kept frozen at -80°C until further analysis.

### Liquid chromatography-tandem mass spectrometry

Prior to the liquid chromatography-tandem mass spectrometry analysis, the dry lipid extracts were dissolved in 200 μl methanol solvent. After vortexing and followed by 10 min centrifugation (11000 rpm at 10°C), the mixed solvents were filtered with 0.2 μm Millipore PTFE syringe filters prior to LC-MS analysis.

A validated Waters Acquity UPLC system, coupled with a Waters Quattro Premier XE triple quadrupole mass spectrometer (Waters, Milford, MA, USA), was used to perform phosphatidylcholine profiling [20]. Reversed-phase chromatography was conducted with an analytic column (BEH C_18,_ 2.1 X 100 mm, 1.7 μm, Waters, Milford, MA, USA) and the binary solvent systems (solvent A: ACN/MeOH (65/35) containing 1% 1.0 M NH_4_Ac and solvent B: 10 mM NH_4_Ac in water with 5% ACN/MeOH (65/35)). Solvent A was programmed to increase from 55% to 70% in 0.1 min, then to 100% in 1.5 min, and hold at 100% for 4.5 min during the gradient elution. The lipid extracts of the tissue and serum (10 μl) were analysed with the column eluting at a constant flow rate of 0.7 mL/min and column temperature at 70°C.

The optimized operation conditions of the tandem mass spectrometer are described as follows. The capillary and cone voltage were set at 2.5 kV and 35 V, respectively, with collision energy of 30 eV. The desolvation temperature was set at 450°C and the ion source temperature at 120°C. Moreover, the desolvation gas was set to a flow rate of 0.7 mL/min and cone gas flow was set to 50 L/h. The system of acquisition range was set from *m/z* 200 to 900. The positive ion electrospray ionization coupled with the precursor ion scan (fixed unique product ion *m/z* = 184) was used to monitor the phosphorylcholine-containing lipids as much as possible.

### Spectral processing

After acquiring spectral data, the raw spectral data were exported into NetCDF in Masslynx V4.1 (Waters, CA, USA). The non-commercial software, MZmine 2.10 [24], was used to process the complex dataset. After a series of the processes, including raw data being imported, peak detection, isotope patterns, peak list alignment, and a gap-filling step, each detected peak signal was normalized to the sum of the total signal response in the phosphorylcholine-containing lipid profile. The normalized dataset was log transformed and mean-center scaled prior to multivariate analysis.

### Identification of phosphorylcholine-containing lipids

**Fig 1:**
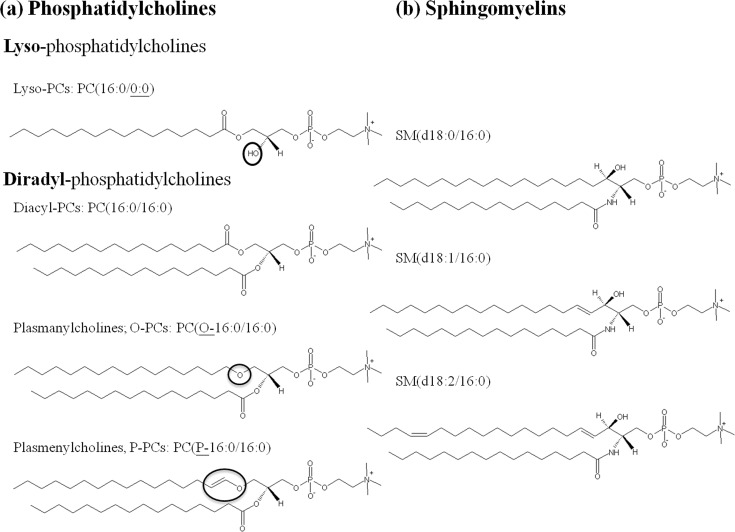
Representative structure of various species of (a) phosphatidylcholines (PCs) and (b) sphingomyelins (SMs).

Phosphorylcholine-containing lipid assignments were referenced to previous studies [20, 25], in-house database, and LIPID MAPS library (http://www.lipidmaps.org). Lyso-PCs were separated from PCs and SMs by the retention time in the chromatogram, whereas SMs were discriminated from PCs via odd m/z based on the nitrogen rule. The fragment patterns in the product ion spectra of [M+H]^+^ were used to determine lyso-PC subclasses. The product ion spectra of [M+Na]^+^ in the ESI positive model as well as [M−Me]^−^ in the ESI negative model were conducted to determine detailed structures of diradyl-PCs (Fig 1). The subclasses of diradyl-PCs, such as diacyl-PCs, alkyl ether-PCs (plasmanylcholines, O-PCs), and vinyl ether-PCs (plasmenylcholines, P-PCs) were discriminated via the product ion spectra of [M+Na]^+^. Once the subclasses of PCs were confirmed, the product ion spectra of [M−Me]^−^, which lost one methyl moiety from the choline head group of PCs, were obtained to infer the composition (including carbon chain length and degree of unsaturation) of fatty acid substituents existing in the PCs. In general, the intensity of the sn-2 carboxylate anion was much higher than the intensity of the sn-1 carboxylate anion in our system. On the other hand, the fragment ion patterns of the [M+Na]^+^ in the ESI positive model were used for identification of SM structure.

### Multivariate analysis

The processed spectral data were examined for lipid effects among groups by partial least-square-discriminate analysis (PLS-DA). PLS-DA, a supervised pattern-recognition technique, is used to examine the internal relationships between the X-variables (metabolites) and response Y (treatments). PLS-DA is a frequently used classification method and is based on the PLS approach [26]. PLS-DA were conducted in Simca 13 software (Umetrics, Umea, Sweden). PLS-DA was performed to determine if the phosphorylcholine-containing lipid profile of different groups could be distinguished. To prevent group over-fitting, the PLS-DA model interpretation quality was assessed with regard to the residuals (R^2^X and R^2^Y). Q^2^ represents the predictive ability parameter, which was also determined with one-seventh excluded for cross-validation. The R^2^ and Q^2^ in the permutated plot depict the fitness of the data and the predictability of the derived model, respectively [27, 28]. The VIP values from validated PLS-DA models were conducted to suggest the crucial lipids for the different clusters or separation patterns. A cut-off threshold, VIP values≥ 1.0 [29], was applied in this study to suggest critical lipids.

The results were then further confirmed by univariate analysis to evaluate the statistical significance of each lipid independently. In this study, the levels of identified lipids were examined with a Kruskal-Wallis test using SPSS 19.0 statistical software (SPSS Inc., Chicago, IL, USA). When a statistical significance (*p*< 0.05) was achieved among groups, all pairwise comparisons, Dunn’s test, were then used for determining statistical significance (with an adjusted *p*< 0.017) between groups.

## Results

### Histopathology

The morphology of the lung epithelium in a naphthalene tolerance model (repeated dose group) showed no obvious differences from the control group, whereas the injury model or non-tolerant model (single dose group) showed the formation of blebs and vacuolization on Clara cells after administering a challenge dose of naphthalene (S1A–S1C Fig). The liver and kidney tissues showed no morphological alterations in either the injury model or the tolerance model when compared with the control group (S1D–S1I Fig).

### Naphthalene induced lipid alterations in the mouse lung

**Fig 2:**
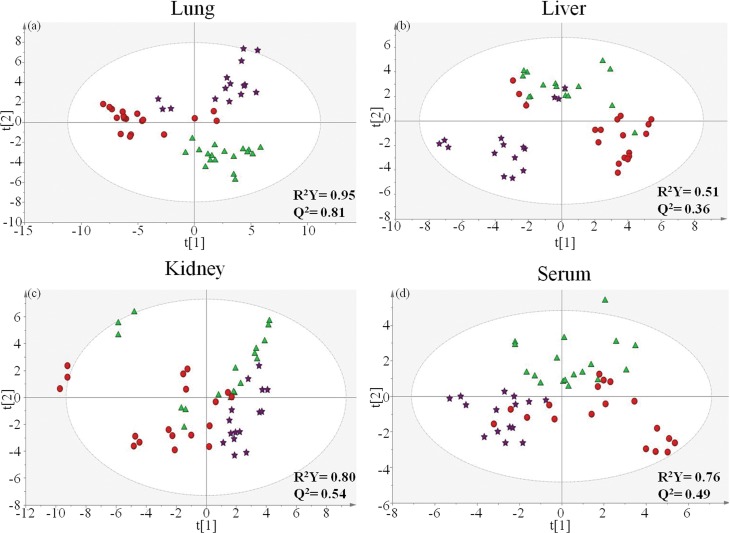
The PLS-DA score plots from the analysis of MS spectra of mouse lungs (a), liver (b), kidneys (c), and serum (d) from naphthalene tolerant model (star), naphthalene injury model (circle), and the control (triangle).

**Fig 3:**
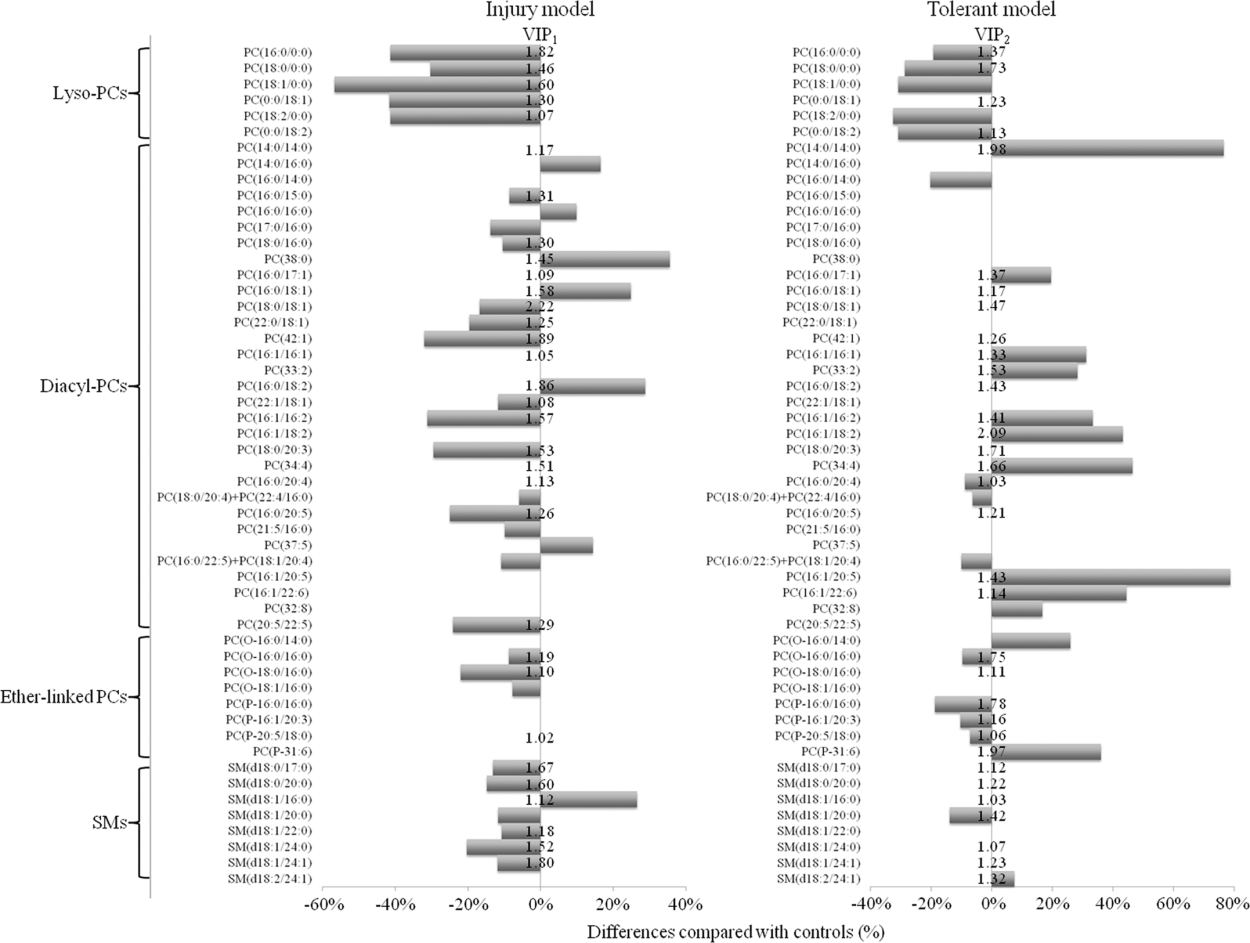
Level changes of mouse lung phosphatidylcholines (PCs) and sphingomyelins (SMs) in the naphthalene-induced injury model and the tolerant model, compared with the control. Listed lipids were passed the statistical threshold (adjusted *p*< 0.05) by Kruskal-Wallis test with Dunn’s test as post hoc analysis in injury or tolerant model. Differences compared with controls (%) <0 or >0 represent decrease or increase of peak area, respectively relative to the control. VIP values >1 from the PLS-DA models were also listed.

The variations of phosphorylcholine-containing lipid profile among three treatments (tolerance, injury, and control) from the analysis of MS spectra on mouse lungs were further analysed in a PLS-DA model (R^2^Y = 0.95; Q^2^ = 0.81). In the scores plot, each spot represents a phosphorylcholine-containing lipid profile from the MS spectrum, which was derived from the triplicate of each biological sample (Fig 2A). The tolerant and control groups were separated from the injury group along the LV1 axis, while the tolerant group was separated from the control group along the LV2 axis. Variable importance in projection (VIP) values over 1 were identified and associated with the separation pattern of tolerant and injury models along the LV1 axis, while additional lipids (VIP> 1) were identified to discriminate between the variance of the tolerant and control groups along the LV2 axis (Fig 3).

In addition to multivariate data analysis, all detected phosphorylcholine-containing lipids were further analysed with a more traditional and conservative approach: Kruskal-Wallis test with Dunn’s test as post hoc analysis to confirm differences of significance (Fig 3 and S1 and S2 Tables). Among the total detected 74 PCs in the mouse lung, 50 PCs showed differences (*p*< 0.017) in either tolerant or injury groups compared with the control group, including lyso-PCs (*n* = 6), diacyl-PCs (n = 30), O-PCs (n = 4), P-PCs (n = 4) and U-PCs (unknown phosphatidylcholines: *n* = 6). There were 14 level differences of SMs over the total 26 detected SMs.

Among six changed lyso-PCs, all of them were decreased (6/6), as shown in Fig 3. Specifically, PC(16:0/0:0), PC(18:0/0:0), PC(18:1/0:0), and PC(18:2/0:0) were lower in both the tolerant and injury models compared with the control group. The levels of PC(0:0/18:1) and PC(0:0/18:2) were also lower in either the tolerant or injury models, respectively, compared with the control group.

Among the changed diacyl-PCs (n = 20) in the injury group, 14 of 20 diacyl-PCs were decreased compared with the control group. However, most changed diacyl-PCs (10/13) were increased in the tolerant group when compared with the control group (Fig 3). Among these 10 diacyl-PCs, eight diacyl-PCs were polyunsaturated fatty acids (PUFAs).

Levels of eight ether-linked PCs, including plasmanylcholine, O-PCs (n = 4) and plasmenylcholines, P-PCs (n = 4), were different in either injured or tolerant animals (Fig 3). Specifically, PC(O-16:0/16:0), PC(O-18:0/16:0) and PC(O-18:1/16:0) were lower in the injury group compared with the control group. On the other hand, PC(P-16:0/16:0), PC(P-16:1/20:3), and PC(P-20:5/18:0) were lower in the tolerant group compared with the control group, while PC(P-31:6) showed an increase in the tolerant group. Finally, there were no obvious patterns among unknown PCs.

Most level differences of SMs were decreased (6/7) in the injury model when compared with the control group, whereas there was no obvious pattern in the tolerant model (Fig 3). Moreover, numerous unknown SMs in both the tolerant and injury model were decreased.

### Naphthalene induced lipid alterations in the mouse liver

One technical replicate of one liver sample from the control group was removed since it is an outlier. The PLS-DA model from the analysis of MS spectra showed variations in the phosphorylcholine-containing lipid profiles among livers from the three groups (tolerance, injury, and control) (R^2^Y = 0.51; Q^2^ = 0.36) (Fig 2B). The tolerant group was separated from the injury group along the LV1 axis, while both tolerant and injury groups were separated from the control group along the LV2 axis.

In addition, a Kruskal-Wallis test with Dunn’s test was conducted to examine the level differences of phosphorylcholine-containing lipids among the livers of three groups. Among the total detected 51 PCs in the liver, nine PCs showed differences after naphthalene exposure, while one SM was different over the total 10 detected SMs (s). There was no obvious trend in each class of lipids in both the tolerant and injury models when compared with the control group. PC(18:0/20:3), PC(20:0/20:4), PC(P-40:2), PC(16:0/0:0), PC(P-20:0/18:2), and PC(P-40:2) were decreased in both the tolerant and injury group when compared with the control group, whereas SM(d18:1/16:0) was increased in the injury group when compared with the control group.

### Naphthalene induced lipid alterations in the mouse kidney

Fig 2C shows the variations in kidney phosphorylcholine-containing lipid profiles among the three groups from the analysis of MS spectra in a PLS-DA model (R^2^Y = 0.80; Q^2^ = 0.54). The tolerant group was separated from the injury group along the LV1 axis, while both the tolerant and injury groups were separated from the control group along the LV2 axis.

After a Kruskal-Wallis test with Dunn’s test, 12 PCs from total detected 42 PCs showed differences after naphthalene exposure, while seven SMs over total 21 detected SMs were different (S1 and S2 Tables). Increased lyso-PCs and diacyl-PCs, such as PC(18:1/0:0), PC(16:0/18:1), PC(18:0/18:1), PC(16:0/20:3), and PC(16:1/22:6), were found in the tolerant group. Increased PC(18:2/0:0), PC(17:0/18:1), and PC(19:0/18:2) were discovered in the injury group. Moreover, ether-linked PCs, PC(O-16:0/16:0) and PC(P-20:5/18:0), were decreased in the tolerant group compared with the control group. Among those significantly changed SMs, most SMs were increased in the injury group compared with the control group.

### Naphthalene induced lipid alterations in the mouse serum

Fig 2D shows the variations in serum phosphorylcholine-containing lipid profiles from the analysis of MS spectra for the three groups in a PLS-DA model (R^2^Y = 0.76; Q^2^ = 0.49). The tolerant group was separated from the injury group along the LV1 axis, while both the tolerant and the injury groups were separated from the control group along the LV2 axis.

After a Kruskal-Wallis test with Dunn’s test, there were nine differences of PCs among the total detected 43 PCs in the serum of treated mice, while there was one difference of SM over the total nine detected SMs (S1 and S2 Tables). PC(16:0/16:1), PC(33:2), PC(17:0/18:2), PC(16:1/18:2), PC(35:3), and PC(16:0/20:5) in the tolerant group as well as PC(33:2), PC(17:0/18:2), PC(18:0/18:2), and PC(35:3) in the injury group were increased when compared with the control group. SM(d18:1/16:0) was decreased in the tolerant group compared with the control group.

## Discussion

In this study, we utilized MS-based lipidomics to explore respiratory toxicity induced by naphthalene by examining lipid alterations between an injury model and tolerant model. Effects on non-susceptible organs (liver and kidney) were also examined. The final goal was to associate changes of lipid profiles with toxic or protective effects of naphthalene-induced injury or tolerance.

From the histopathological results of mouse lung, the tolerant group showed almost no differences compared to the control group. However, with the single dose of the naphthalene group, the morphological changes, such as vacuolated and swollen Clara cells, were obvious. These morphological findings were similar to results from previous naphthalene studies [11]. These membrane morphological variances may be due to the alterations of phosphorylcholine-containing lipids in the lung, which are involved with membrane integrity and membrane function. Even though there were no histological variances in the non-target organs, livers and kidneys, phosphorylcholine-containing lipid profiling of those organs were still observed.

The effects of naphthalene treatments on phosphorylcholine-containing lipid profiling among the lung, liver, and kidney were varied. The lung phosphorylcholine-containing lipids appeared more susceptible to naphthalene treatments compared with those in the liver, kidney, and serum. Higher percentages of the individual phosphorylcholine-containing lipid species were altered in the mouse lung compared with other sample types. The changes in lipid profiles associated with the perturbations caused by naphthalene in injury or tolerant models are illustrated in the following sections.

Our results showed the levels of lyso-PCs in the lungs, including PC(16:0/0:0), PC(18:0/0:0), PC(18:1/0:0), PC(0:0/18:1), PC(18:2/0:0), and PC(0:0/18:2), were all decreased in both the tolerant group and injury group when compared with the control group, whereas there was little variance in the liver, kidney, and serum. Lyso-PCs, which are linked to the fatty-acyl chain at position sn-1 or sn-2 on the glycerol backbone (Fig 1), are one of the structural components of cell membranes. The phospholipase A_2_ (PLA_2_) produce the sn-1 lyso-PCs, and the phospholipase A_1_ (PLA_1_) produce the sn-2 lyso-PCs [30]. The sn-1 lyso-PCs are the major pattern in the lung generated from the PCs in the cell membrane or lipoproteins [31]. After exposure to naphthalene, decreased lyso-PCs could be due to the dysfunction of phospholipase activity. On the other hand, the decreased levels of lyso-PCs were also related to reduce the elastic free energy so that the membrane can progress in the ability to bend to maintain membrane stability as the curvature suppresses [32]. The changes in lyso-PCs could be a signature as an important factor that may disturb cell function and signalling by promoting positive curvature of the membrane [32, 33]. Additionally, lyso-PCs also played a role to facilitate the hydrophilic channel (membrane pore) formation based on their inverted cone molecular shape [34]. Thus, the reduction of lyso-PCs may indicate the protective mechanism to avoid the formation of pores on the membrane. Even though no morphological changes were observed in the tolerant model, some subtle lyso-PC perturbations still occurred. Potential long-term structure and function dysfunction is suspected.

Our results showed opposite trends for diacyl-PC levels between injury and tolerant mice, which may reflect different consequences of the membrane states affected by different naphthalene treatments. There was an increasing trend for most diacyl-PCs in the lungs of tolerant mice, while most showed a decreasing trend in mouse diacyl-PC profiles in the lungs of injured mice. Diacyl-PCs played an important role in membrane stability and flexibility against oxidative stress and ameliorating cell/organelle swelling and tension-induced leakage [35, 36]. Thus, the increasing trend of diacyl-PCs in the tolerance model may imply that low-level naphthalene treatment adoption prior to high challenge treatment induces a high level of diacyl-PCs to strengthen the physico-chemical properties of cell membrane to provide more capacity for membrane morphological alterations to ameliorate the naphthalene-induced molecular perturbation. Inversely, the decreasing trends of diacyl-PCs in the naphthalene injury model may reflect a weaker or damaged membrane structure state under oxidative insult from the challenge dose of naphthalene.

On the other hand, three plasmanylcholine were decreased in the lungs of the injury group compared with the control group in this study. Previous studies showed that plasmanylcholine was related to the regulation of fluidity of the cell membrane, facilitating membrane fusion, the release of PUFA, anti-oxidant function, and energy storage [15, 37]. Exposure to a single dose of naphthalene disrupted the membrane integrity; nevertheless, repeated injections of naphthalene appeared to produce insignificant alternations in our morphological results. These results may indicate that decreasing levels of plasmanylcholine might play a physiological role in naphthalene-induced membrane injury. These alterations of diacyl-PC and O-PC profiles provide more molecular information for discussing the variances between the morphological results of naphthalene tolerance and injury models.

In this study, there were eight of ten increased diacyl-PCs consisting of PUFAs, including PC(16:1/16:1), PC(33:2), PC(16:1/16:2), PC(16:1/18:2), PC(34:4), PC(16:1/20:5), PC(16:1/22:6), and PC(32:8), in the tolerant group over the control group. Previous studies also mentioned that polyunsaturated PCs enabled enlarging of the membrane surface to possess greater capacity to endure swelling of the cells and organelles [38, 39]. Additionally, a higher degree of unsaturation PCs leads to an inherent proclivity towards an upper grading of conformational flexibility in the membrane architecture [40]. Therefore, the increase of PCs consisting of PUFAs in the tolerant mice may indicate a phenomenon of strength in the cell membrane integrity and cellular function to resist further naphthalene induced-injury in the lungs of tolerant mice. PUFAs may also act as anti-oxidants to attenuate naphthalene-induced molecular perturbation.

Due to the vinyl ether bond of plasmenylcholine, plasmenylcholine was more susceptible to oxidation stress and was connected to anti-oxidant properties [41]. The decreasing level of three P-PCs, including PC(P-16:0/16:0), PC(P-16:1/20:3), and PC(P-38:5) in the tolerant group compared with the control group may imply that the consumption of sacrificial oxidants or scavengers against the naphthalene induced molecular perturbations to protect other membrane components. A similar reduction trend of P-PCs was also observed in the lungs of rats chronically exposed to ambient fine particulate matter [21]. In our previous study [14], an significant increase in acetone in the bronchoalveolar lavage fluid (BALF) from the same injured mice was found. Acetone is one of the ketone bodies that is generated from the lipolysis or lipid peroxidation [42]. The increase in acetone in the BALF of the injured mice may reflect lipid peroxidation in the airways of the injured mice compared with the tolerant mice [14]. Previous studies have also reported the presence of oxidative damage and lipid oxidation after naphthalene treatment in the rodent models and cell cultures [43–46].

SMs are the precursors of bioactive cellular signal molecules such as ceramides, which may trigger cell proliferation, rejuvenation, and apoptosis, as well as possibly being involved with inflammation processes. The accumulation of ceramides has been recorded in several lung-related injuries and disorders [15, 47]. Additionally, a study suggested an anti-inflammatory role for SMs [48]. Thus, the reduction of most SM species in the injury group compared with the control group may cause accumulation of ceramides, which then activate a series of abnormal cellular activities, and pulmonary inflammation, which was observed in previous lung related disorders or diseases [19, 49].

There were fewer naphthalene-induced phosphorylcholine-containing lipid alterations in the mouse liver despite treatments, compared with those in the lungs. Less impact on liver phosphorylcholine-containing lipids could be attributed to the sufficient glutathione pool in the liver to detoxify reactive naphthalene metabolites. Previous literature showed that the glutathione pool played a role in cellular defence to inactivate reactive intermediates and prevent lipid peroxidation [50]. Moreover, our previous metabolomics study using the same animal models showed that glutathione synthesis might be increased in the liver of tolerant mice to protect cells from reactive naphthalene metabolites [14].

Even though no morphological alteration was observed in the kidney after naphthalene treatments, few phosphorylcholine-containing lipid alterations were observed in the mouse kidney despite treatments. The increasing trends of diacyl-PCs and SMs in the kidneys of injury or tolerant mice compared with the control group were contrary to the trends in the mouse lungs, which may be due to tissue specificity. There was only one plasmanylcholine, and one plasmenylcholine decreased in the tolerant group compared with the control group. Plasmanylcholine was associated with regulating the cell membrane fluidity and mobility; in addition, plasmenylcholine may function as an anti-oxidant [51]. The depletion of plasmanylcholine and plasmenylcholine in the tolerant group may be an adaption or detoxifying mechanism in the kidney.

Most SMs were increased in the kidneys of injured mice. Although there was no strong evidence of naphthalene inducing kidney injury in mice, a study reported a close relationship between the lung and kidney involving health and disease [52]. Another study showed that increased levels of certain SM promoted ATP and lactate production via glycolysis and then activated inflammation, which might play a pivotal role in the progression of diabetics in the kidney [53]. Thus, the increasing trend of most kidney SMs in this study may be involved with disturbed energetic pathways and induced inflammation in the mouse kidney after naphthalene insult. Our previous NMR-based metabolomic results also showed accumulation of lactate, which may be viewed as an abnormal energetic homeostasis in the kidneys of single-dose treated mice [14].

Blood is a homeostasis pool, which can collect molecular changes from different organs and replenish the elements back to the organs or tissue. In this study, we aimed to examine whether lipid alterations in blood may reflect respiratory toxicity for application in the human population. Only few PCs and SMs were different in the mouse serum despite treatment methods. Consistently, decreased PC(18:0/0:0) and increased PC(33:2) were both observed in the lung and serum samples in the tolerance model comparing with the control group. A previous study has suggested that decreased levels of lyso-PCs, such as PC(18:0/0:0), in the plasma were related to cell proliferation in patients with lung cancer [54]. Whether PC(18:0/0:0) is a biomarker for pulmonary abnormalities needs further evaluation. Moreover, we found increased PC(16:0/18:1) only in the lungs of injured mice but not tolerant mice. Interestingly, decreased PC(16:0/18:1) was discovered in the serum of injured mice but not tolerant mice. PC(16:0/18:1) is a component of the mammalian pulmonary surfactant system, which maintain respiratory function. Previous studies showed that increased levels of PC(16:0/18:1) in the bronchoalveolar lavage fluid were observed in children with respiratory infections or acute lung injury [55, 56]. Thus, PC(16:0/18:1) may play an important role in maintaining pulmonary function. The contrary levels of PC(16:0/18:1) between target organ-lung and system response-serum needs more study to confirm whether the lipids in the serum reflect lung injury.

Increased PC(18:0/18:2) was found both in the liver and serum samples of the injury model compared with the control group, while there were no consistent alteration patterns between lipids from the kidney and serum. In general, there are more similar trends of lipid alteration between the liver and serum, which may be due to the liver being the major contributor of blood components.

In this study, we aimed to understand the changes in lipid profiles associated with the perturbations caused by naphthalene in the respiratory system by examining phosphorylcholine-containing lipid alterations. Our analytical platform only focused on examining PC and SM species. The expansion of our current platform for analysis of more lipid classes, such as triacylglycerols is in progress. On the other hand, future studies examining changes in overall lipid profile, including triacylglycerols, phospholipids, sphingolipids, and cholesterols using quadrupole time-of-flight mass spectrometer (q-TOF) can assist in understanding of naphthalene tolerance or toxicity more completely.

Our current platform is unable to distinguish ω-3 and ω-6 PUFAs. The ω-3 and ω-6 PUFAs were two major derivatives of PUFAs in the cell membrane that possess diverse bioactivities. Therefore, the ratio of ω-3s to ω-6s was suggested to be a predictor of several diseases [57]. In addition, the ω-3s, such as eicosapentaenoic acid and docosahexaenoic acid, have been involved with several beneficial effects for human health, including the prevention of cardiovascular disease and cancer suppression [57]. An additional platform to distinguish ω-3 and ω-6 lipids can assist with understanding the roles of lipids on pulmonary toxicity.

From our results, the weak tendency for lipid changes between the lung and serum after naphthalene treatment might be attributed to the lung not being a major blood provider, resulting in the lipid effects from lungs recorded in the blood being diluted by other organs. Since the disturbance of serum lipids might be contributed to multiple organs, further studies to confirm whether PC(16:0/18:1) is a biomarker for pulmonary injury are needed.

Assessment of molecular responses in BALF can reflect molecular changes more directly in airway epithelial cells than in whole lungs. Moreover, BALF sampling is relatively less invasive compared with the lung. Application of lipidomics in order to study lipid changes in the BALF to be able to associate these changes with respiratory toxicity or diseases has been reported [58, 59]. Recently, our lab has optimised our MS platform to analyse lipid changes in the BALF in response to naphthalene in another naphthalene toxicity study. The results of lipid changes in the BALF will be further compared with those from the lung or the alveolar regions to reveal site specific lipid responses in the respiratory system.

## Conclusions

Naphthalene-induced respiratory toxicity or tolerance was correlated with changes in phosphorylcholine-containing lipids were suggested by our previous ^1^H NMR-based metabolomic approach. In this study, we examined alternations of phosphorylcholine-containing lipids in the lung, liver, kidneys, and serum by UPLC-MS/MS in order to understand naphthalene toxicity or tolerance in target, non-target organs, and biofluids. Fewer phosphorylcholine-containing lipid effects of naphthalene treatments were found in the liver and kidneys compared to the lung. Higher ratios of phosphorylcholine-containing lipids were altered in the lungs of naphthalene treated mice than those in other organs, demonstrating that the lung is more susceptible to naphthalene. Naphthalene-induced respiratory toxicity or tolerance is correlated with lipid perturbation. In the lungs of tolerant mice, strengthened membrane flexibility by increases of diacyl-PCs and consumption of anti-oxidative relating lipids, such as P-PCs, were observed against naphthalene-induced molecular perturbation. On the other hand, the alteration of lyso-PCs and SMs could reflect perturbed cell function and pulmonary inflammation in the lungs of injured mice. The alteration of phosphorylcholine-containing lipid fingerprints in the mouse lungs of our tolerant model (7 day period) illustrated several protective roles to against naphthalene insults in this study. However, the effects of chronic naphthalene exposure have shown the carcinogenic potentials in animal models. More studies are warranted to provide more evidences for investing the variety of naphthalene-mediated responses. Further examination of the critical lipids related to pulmonary injury in serum will help us to understand the information on pulmonary toxicity for application in the human population.

## Supporting information

S1 Fig Histopathological results of the lungs, liver, and kidneys from ICR mice after different naphthalene treatments. (a) (d) (g) **Control** was intraperitoneal administered with olive oil daily for eight days, (b) (e) (h) **Injury** model was intraperitoneal administered with vehicle (olive oil) daily for seven days, followed by administered a challenged dose (300 mg/kg naphthalene) on the eighth day, and (c) (f) (i) **Tolerant** model was intraperitoneal administered with 200 mg/kg naphthalene daily for seven days, followed by administered a challenged dose (300 mg/kg naphthalene) on the eighth day. The formation of the vacuoles (★) in the non-ciliated epithelial cell (Clara cell) were observed in the injury model.
(TIF)

S1 Table Level changes of the all detected phosphatidylcholines in the lungs, liver, kidneys, and serum from mice receiving different naphthalene treatments compared to the controls. O-PCs: alkyl ether-phosphatidylcholines (plasmanylcholines); P-PCs: vinyl ether-phosphatidylcholines (plasmenylcholines); U-PCs: unknown phosphatidylcholines.
^a^ Fold changes> 1 or < 1 represent increase or decrease of peak area, respectively relative to its corresponding.
**Tolerant** model was intraperitoneal administered with 200 mg/kg naphthalene daily for seven days, followed by administered a challenged dose (300 mg/kg naphthalene) on the eighth day. **Injury** model was intraperitoneal administered with vehicle (olive oil) daily for seven days, followed by administered a challenged dose (300 mg/kg naphthalene) on the eighth day. **Control (C)** group was intraperitoneal administered with olive oil daily for eight days.
* The significant differences (adjusted *p*< 0.05) of the identified lipids by Kruskal-Wallis test with Dunn’s test as post hoc analysis.
“-”was representative “not detected”.
(DOC)

S2 Table Level changes of the all detected sphingomyelins in the lungs, liver, kidneys, and serum from mice receiving different naphthalene treatments compared to the controls. U-SMs: unknown sphingomyelins.
^a^ Fold changes> 1 or < 1 represent increase or decrease of peak area, respectively relative to its corresponding.
**Tolerant** model was intraperitoneal administered with 200 mg/kg naphthalene daily for seven days, followed by administered a challenged dose (300 mg/kg naphthalene) on the eighth day. **Injury** model was intraperitoneal administered with vehicle (olive oil) daily for seven days, followed by administered a challenged dose (300 mg/kg naphthalene) on the eighth day. **Control (C)** group was intraperitoneal administered with olive oil daily for eight days.
* The significant differences (adjusted *p*< 0.05) of the identified lipids by Kruskal-Wallis test with Dunn’s test as post hoc analysis.
“-”was representative “not detected”.
(DOC)

S1 File The raw and normalized dataset for multivariate analysis. (XLSX)

S2 File The raw and normalized dataset for univariate analysis. (XLSX)
