# Supplementary figures and images for: Mass spectrometry-based lipidomics to explore the biochemical effects of naphthalene toxicity or tolerance in a mouse model
Source: PLoS One. 2018 Oct 1;13(10):e0204829. doi: 10.1371/journal.pone.0204829 (PMC6166967; doi:10.1371/journal.pone.0204829)

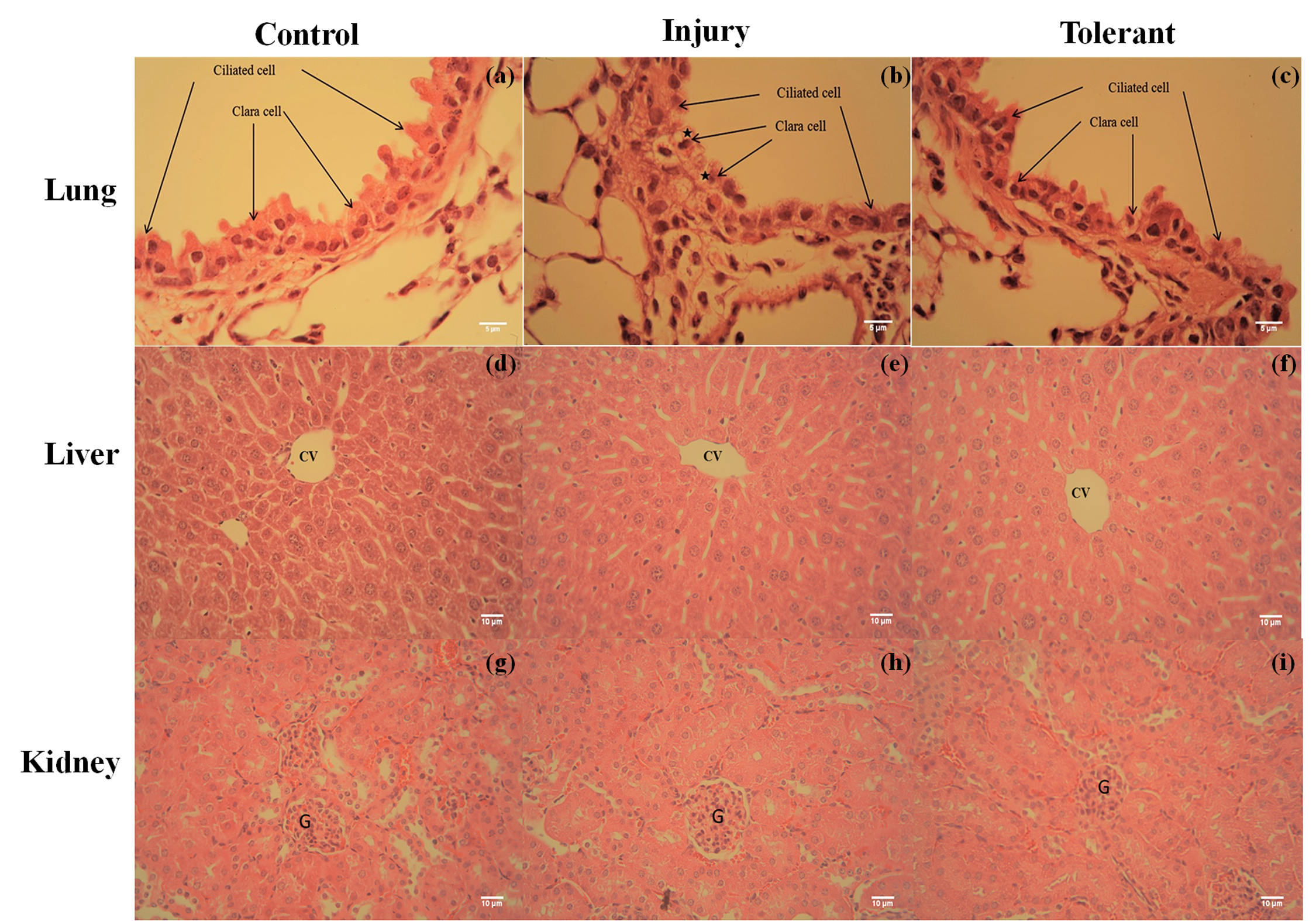

Supplement: S1 Fig — (a) (d) (g) Control was intraperitoneal administered with olive oil daily for eight days, (b) (e) (h) Injury model was intraperitoneal administered with vehicle (olive oil) daily for seven days, followed by administered a challenged dose (300 mg/kg naphthalene) on the eighth day, and (c) (f) (i) Tolerant model was intraperitoneal administered with 200 mg/kg naphthalene daily for seven days, followed by administered a challenged dose (300 mg/kg naphthalene) on the eighth day. The formation of the vacuoles (★) in the non-ciliated epithelial cell (Clara cell) were observed in the injury model. (TIF) [file pone.0204829.s001.tif]
