# Supplementary material for: Mass spectrometry-based lipidomics to explore the biochemical effects of naphthalene toxicity or tolerance in a mouse model
Source: PLoS One. 2018 Oct 1;13(10):e0204829. doi: 10.1371/journal.pone.0204829 (PMC6166967; doi:10.1371/journal.pone.0204829)
Supplement: S1 Table — O-PCs: alkyl ether-phosphatidylcholines (plasmanylcholines); P-PCs: vinyl ether-phosphatidylcholines (plasmenylcholines); U-PCs: unknown phosphatidylcholines. a Fold changes> 1 or < 1 represent increase or decrease of peak area, respectively relative to its corresponding. Tolerant model was intraperitoneal administered with 200 mg/kg naphthalene daily for seven days, followed by administered a challenged dose (300 mg/kg naphthalene) on the eighth day. Injury model was intraperitoneal administered with vehicle (olive oil) daily for seven days, followed by administered a challenged dose (300 mg/kg naphthalene) on the eighth day. Control (C) group was intraperitoneal administered with olive oil daily for eight days. * The significant differences (adjusted p< 0.05) of the identified lipids by Kruskal-Wallis test with Dunn’s test as post hoc analysis. “-”was representative “not detected”. (DOC) [file pone.0204829.s002.doc]

**S1 Table.** Level changes of the all detected phosphatidylcholines in the lungs, liver, kidneys, and serum from mice receiving different naphthalene treatments compared to the controls

|  | **Fold changes ^a^** | | | | | | | |
| --- | --- | --- | --- | --- | --- | --- | --- | --- |
|  | **Lung** | | **Liver** | | **Kidney** | | **Serum** | |
| **Lipids** | Injury/C | Tolerant/C | Injury/C | Tolerant/C | Injury/C | Tolerant/C | Injury/C | Tolerant/C |
| **Lyso-PCs** | n= 6 | | n= 6 | | n= 5 | | n= 4 | |
| PC(16:0/0:0) | 0.59* | 0.81* | 0.64* | 1.02 | 1.09 | 1.09 | 1.00 | 1.00 |
| PC(18:0/0:0) | 0.70* | 0.71* | 0.65 | 0.79 | 1.13 | 0.99 | 1.05 | 0.83* |
| PC(18:1/0:0) | 0.43* | 0.69* | 0.73 | 1.18 | 1.21 | 1.36* | 0.82 | 1.09 |
| PC(0:0/18:1) | 0.58* | 0.89 | - | - | - | - | - | - |
| PC(18:2/0:0) | 0.59* | 0.67* | - | - | 1.53* | 1.29 | 0.79 | 0.89 |
| PC(0:0/18:2) | 0.75 | 0.69* | 0.85 | 0.80 | - | - | - | - |
| PC(20:4/0:0) | - | - | 0.78 | 0.69 | - | - | - | - |
| PC(0:0/22:6) | - | - | 0.76 | 0.80 | 0.97 | 1.02 | - | - |
| **Diacyl-PCs** | n= 42 | | n= 37 | | n= 24 | | n= 33 | |
| PC(14:0/14:0) | 0.90 | 1.77* | - | - | - | - | - | - |
| PC(14:0/16:0) | 1.17* | 1.13 | - | - | - | - | - | - |
| PC(16:0/14:0) | 0.97 | 0.80* | - | - | - | - | - | - |
| PC(16:0/15:0) | 0.91* | 1.05 | - | - | - | - | - | - |
| PC(16:0/16:0) | 1.10* | 0.93 | 1.13 | 1.08 | 0.79* | 0.93 | 1.00 | 0.96 |
| PC(17:0/16:0) | 0.86* | 0.95 | 0.99 | 1.04 | - | - | - | - |
| PC(18:0/16:0) | 0.90* | 0.96 | 1.10 | 0.91 | 0.93 | 0.92 | - | - |
| PC(34:0) | - | - | - | - | - | - | 0.82 | 0.75 |
| PC(38:0) | 1.36* | 1.02 | - | - | - | - | 0.98 | 1.04 |
| PC(16:0/16:1) | 1.11 | 0.96 | 0.66 | 1.39 | 0.65 | 1.12 | 0.86 | 1.78* |
| PC(16:0/17:1) | 1.02 | 1.20* | 0.89 | 1.30 | - | - | 1.06 | 1.45 |
| PC(16:0/18:1) | 1.25* | 1.08 | 0.96 | 1.21 | 1.07 | 1.08* | 0.79* | 1.00 |
| PC(17:0/18:1) | 0.98 | 0.98 | 0.95 | 1.10 | 1.23* | 1.05 | 1.13 | 0.97 |
| PC(18:0/18:1) | 0.83* | 1.01 | 0.78 | 0.98 | 1.12* | 1.13* | 1.04 | 1.06 |
| PC(18:1/19:0) + PC(16:0/21:1) | 0.95 | 1.08 | - | - | - | - | - | - |
| PC(38:1) | - | - | - | - | - | - | 1.03 | 1.10 |
| PC(22:0/18:1) | 0.80* | 1.09 | - | - | - | - | - | - |
| PC(40:1) | - | - | - | - | - | - | 1.16 | 0.88 |
| PC(42:1) | 0.68* | 0.98 | - | - | - | - | - | - |
| PC(16:1/16:1) | 0.93 | 1.31* | - | - | - | - | - | - |
| PC(33:2) | 1.11 | 1.28* | - | - | - | - | 1.24* | 1.37* |
| PC(16:0/18:2) | 1.29* | 1.12 | 1.09 | 1.02 | 0.93 | 1.04 | 1.04 | 1.00 |
| PC(17:0/18:2) | - | - | 1.09 | 1.01 | - | - | 1.22* | 1.24* |
| PC(18:0/18:2) | 1.07 | 0.99 | 1.15* | 1.02 | 1.07 | 1.07 | 1.13* | 1.03 |
| PC(19:0/18:2) | - | - | 0.62* | 0.78 | 1.20* | 1.11 | 1.02 | 0.93 |
| PC(18:0/20:2) + PC(20:0/18:2) | 0.95 | 0.91 | - | - | - | - | 0.78 | 0.67 |
| PC(22:1/18:1) | 0.88* | 1.04 | - | - | - | - | - | - |
| PC(16:1/16:2) | 0.69* | 1.33* | - | - | - | - | - | - |
| PC(16:1/18:2) | 1.04 | 1.43* | 0.78 | 1.22 | 1.09 | 1.27 | 1.35 | 2.60* |
| PC(17:1/18:2) | - | - | 1.27* | 1.23 | - | - | - | - |
| PC(35:3) | - | - | - | - | - | - | 1.68* | 1.68* |
| PC(16:0/20:3) | 1.04 | 0.96 | 0.92 | 1.08 | 0.82 | 1.16* | 1.06 | 1.11 |
| PC(18:0/20:3) | 0.71* | 0.76 | 0.75* | 0.84* | 0.86 | 0.94 | 0.93 | 1.02 |
| PC(40:3) | - | - | - | - | - | - | 0.97 | 0.96 |
| PC(34:4) | 0.84 | 1.47* | 0.86 | 1.64 | - | - | - | - |
| PC(35:4) | - | - | 1.01 | 1.02 | - | - | - | - |
| PC(18:2/18:2) | - | - | 1.21 | 1.17 | - | - | - | - |
| PC(16:0/20:4) | 1.01 | 0.91* | 1.04 | 0.98 | 0.87 | 0.87 | 0.92 | 1.01 |
| PC(37:4) | - | - | 0.88 | 1.09 | - | - | 1.11 | 1.04 |
| PC(18:0/20:4)+PC(22:4/16:0) | 0.94* | 0.94* | 1.08 | 0.87 | 0.99 | 0.99 | 1.00 | 0.95 |
| PC(19:0/20:4) | - | - | 0.97 | 0.79 | - | - | - | - |
| PC(18:0/22:4) | - | - | - | - | 1.10 | 0.94 | - | - |
| PC(20:0/20:4) | 1.11 | 1.01 | 0.76 | 0.53* | - | - | - | - |
| PC(16:0/19:5) | 0.93 | 0.88 | - | - | - | - | - | - |
| PC(16:0/20:5) | 0.75* | 1.02 | 0.72 | 1.12 | 0.87 | 1.19 | 0.85 | 1.63* |
| PC(21:5/16:0) | 0.90* | 0.96 | - | - | - | - | - | - |
| PC(37:5) | 1.14* | 1.01 | - | - | - | - | - | - |
| PC(16:0/22:5)+PC(18:1/20:4) | 0.89* | 0.90* | 1.05 | 1.13 | 0.95 | 1.03 | 1.05 | 0.95 |
| PC(22:5/18:0) | 1.88 | 1.70 | - | - | - | - | 0.93 | 0.81 |
| PC(42:5) | - | - | - | - | - | - | 0.95 | 0.86 |
| PC(16:1/20:5) | 1.15 | 1.79* | 0.86 | 1.68 | 1.01 | 1.14 | - | - |
| PC(37:6) | - | - | 0.99 | 0.91 | - | - | - | - |
| PC(16:0/22:6) | - | - | 1.09 | 1.01 | 0.92 | 0.97 | 0.91 | 0.85 |
| PC(39:6) | - | - | 1.19 | 1.00 | - | - | - | - |
| PC(18:0/22:6) | 0.96 | 1.00 | 1.19 | 0.95 | 0.90 | 0.91 | 0.95 | 0.79 |
| PC(41:6) | - | - | 0.88 | 0.93 | - | - | - | - |
| PC(42:6) | - | - | 0.65 | 0.61 | - | - | - | - |
| PC(16:1/22:6) | 1.08 | 1.45* | 0.85 | 1.23 | 0.84 | 1.34* | 1.04 | 1.71 |
| PC(18:1/22:6) | 1.06 | 0.98 | 1.25* | 1.13 | 0.95 | 0.99 | 0.91 | 1.13 |
| PC(32:8) | 1.03 | 1.17* | - | - | - | - | - | - |
| PC(20:4/20:4) | - | - | 1.13 | 0.73 | 1.05 | 1.16 | 1.16 | 1.10 |
| PC(20:5/22:5) | 0.76* | 0.95 | 1.08 | 0.83 | 1.05 | 0.99 | - | - |
| **O-PCs** | n= 6 | | n= 2 | | n= 2 | | n= 2 | |
| PC(O-16:0/14:0) | 0.95 | 1.26* | - | - | - | - | - | - |
| PC(O-16:0/16:0) | 0.91* | 0.90* | 0.86 | 0.91 | 0.88 | 0.78* | - | - |
| PC(O-18:0/16:0) | 0.78* | 0.82 | 0.86 | 0.90 | 0.96 | 0.92 | 1.29 | 0.94 |
| PC(O-18:1/16:0) | 0.92* | 1.00 | - | - | - | - | - | - |
| PC(O-16:0/18:2) | 1.15 | 1.11 | - | - | - | - | 1.14 | 1.01 |
| PC(O-18:1/18:1) | 1.00 | 0.98 | - | - | - | - | - | - |
| **P-PCs** | n= 10 | | n= 3 | | n= 4 | | n= 3 | |
| PC(P-16:0/16:0) | 0.87 | 0.81* | - | - | - | - | - | - |
| PC(P-20:0/16:0) | 0.89 | 0.81 | - | - | - | - | - | - |
| PC(P-16:0/18:1) | 0.95 | 1.00 | - | - | 1.21 | 1.12 | - | - |
| PC(P-20:0/18:2) | - | - | 0.80* | 0.91 | - | - | - | - |
| PC(P-40:2) | - | - | 0.62* | 0.71* | - | - | - | - |
| PC(P-16:0/20:3) | 0.85 | 0.78 | 1.26 | 1.05 | 1.21 | 0.92 | 1.16 | 0.86 |
| PC(P-38:3) | 1.06 | 0.95 | - | - | - | - | - | - |
| PC(P-16:1/20:3) | 1.09 | 0.90* | - | - | - | - | 0.60 | 0.53 |
| PC(P-20:5/18:0) | 1.01 | 0.93* | - | - | 0.79 | 0.66* | 1.33 | 0.77 |
| PC(P-31:6) | 1.10 | 1.36* | - | - | - | - | - | - |
| PC(P-38:6) | 0.96 | 1.03 | - | - | 1.09 | 0.91 | - | - |
| PC(P-16:1/22:5) | 0.94 | 0.93 | - | - | - | - | - | - |

O-PCs: alkyl ether-phosphatidylcholines (plasmanylcholines); P-PCs: vinyl ether-phosphatidylcholines (plasmenylcholines).

^a^ Fold changes> 1 or < 1 represent increase or decrease of peak area, respectively relative to its corresponding.

**Tolerant** model was intraperitoneal administered with 200 mg/kg naphthalene daily for seven days, followed by administered a challenged dose (300 mg/kg naphthalene) on the eighth day. **Injury** model was intraperitoneal administered with vehicle (olive oil) daily for seven days, followed by administered a challenged dose (300 mg/kg naphthalene) on the eighth day. **Control (C)** group was intraperitoneal administered with olive oil daily for eight days.

* The significant differences (adjusted *p*< 0.05) of the identified lipids by Kruskal-Wallis test with Dunn’s test as post hoc analysis.

“-“ was representative “not detected”.
