# Supplementary material for: Mass spectrometry-based lipidomics to explore the biochemical effects of naphthalene toxicity or tolerance in a mouse model
Source: PLoS One. 2018 Oct 1;13(10):e0204829. doi: 10.1371/journal.pone.0204829 (PMC6166967; doi:10.1371/journal.pone.0204829)
Supplement: S2 Table — U-SMs: unknown sphingomyelins. a Fold changes> 1 or < 1 represent increase or decrease of peak area, respectively relative to its corresponding. Tolerant model was intraperitoneal administered with 200 mg/kg naphthalene daily for seven days, followed by administered a challenged dose (300 mg/kg naphthalene) on the eighth day. Injury model was intraperitoneal administered with vehicle (olive oil) daily for seven days, followed by administered a challenged dose (300 mg/kg naphthalene) on the eighth day. Control (C) group was intraperitoneal administered with olive oil daily for eight days. * The significant differences (adjusted p< 0.05) of the identified lipids by Kruskal-Wallis test with Dunn’s test as post hoc analysis. “-”was representative “not detected”. (DOC) [file pone.0204829.s003.doc]

**S2 Table.** Level changes of the all detected sphingomyelins in the lungs, liver, kidneys, and serum from mice receiving different naphthalene treatments compared to the controls

|  | **Fold changes ^a^** | | | | | | | |
| --- | --- | --- | --- | --- | --- | --- | --- | --- |
|  | **Lung** | | **Liver** | | **Kidney** | | **Serum** | |
| **Lipids** | Injury/C | Tolerant/C | Injury/C | Tolerant/C | Injury/C | Tolerant/C | Injury/C | Tolerant/C |
| **SMs** | n= 13 | | n= 5 | | n= 9 | | n= 6 | |
| SM(d18:0/17:0) | 0.87* | 1.05 | - | - | - | - | - | - |
| SM(d18:0/20:0) | 0.85* | 0.95 | - | - | - | - | - | - |
| SM(d18:0/22:0) | 8.27 | 1.19 | - | - | - | - | - | - |
| SM(d18:0/24:1) | 10.21 | 1.07 | - | - | - | - | - | - |
| SM(d18:1/16:0) | 1.27* | 0.86 | 1.55* | 1.01 | 0.86 | 1.01 | 1.00 | 0.77* |
| SM(d18:1/20:0) | 0.88* | 0.86* | - | - | 1.24* | 1.19 | - | - |
| SM(d18:1/22:0) | 0.89* | 0.97 | 1.07 | 0.72 | 1.23* | 1.14 | 0.96 | 0.71 |
| SM(d18:1/24:0) | 0.80* | 1.20 | 1.14 | 0.74 | 1.31* | 1.14 | 1.01 | 0.78 |
| SM(d18:1/22:1) | 0.96 | 1.09 | - | - | 1.23* | 1.03 | - | - |
| SM(d18:1/24:1) | 0.88* | 1.03 | 1.10 | 0.99 | 1.13* | 1.16 | 1.02 | 0.87 |
| SM(d18:2/16:0) | 0.94 | 1.01 | - | - | 0.89 | 1.02 | 1.13 | 0.97 |
| SM(d18:2/23:0) | 0.92 | 0.92 | 1.14 | 0.95 | 1.49* | 1.12 | - | - |
| SM(d18:2/24:1) | 0.95 | 1.07* | - | - | 1.10 | 1.08 | 1.18 | 0.86 |

^a^ Fold changes> 1 or < 1 represent increase or decrease of peak area, respectively relative to its corresponding.

**Tolerant** model was intraperitoneal administered with 200 mg/kg naphthalene daily for seven days, followed by administered a challenged dose (300 mg/kg naphthalene) on the eighth day. **Injury** model was intraperitoneal administered with vehicle (olive oil) daily for seven days, followed by administered a challenged dose (300 mg/kg naphthalene) on the eighth day. **Control (C)** group was intraperitoneal administered with olive oil daily for eight days.

* The significant differences (adjusted *p*< 0.05) of the identified lipids by Kruskal-Wallis test with Dunn’s test as post hoc analysis.

“-“ was representative “not detected”.
